# Staged Endovascular Treatment for Symptomatic Occlusion Originating From the Intracranial Vertebral Arteries in the Early Non-acute Stage

**DOI:** 10.3389/fneur.2021.673367

**Published:** 2021-06-16

**Authors:** Hongzhou Duan, Li Chen, Shengli Shen, Yang Zhang, Chunwei Li, Zhiqiang Yi, Yingjin Wang, Jiayong Zhang, Liang Li

**Affiliations:** ^1^Department of Neurosurgery, Peking University First Hospital, Beijing, China; ^2^Department of Pediatric Cardiology, Beijing Anzhen Hospital, Capital Medical University, Beijing, China

**Keywords:** occlusion, endovascular treatment, recanalization, vertebral artery, staged

## Abstract

**Background:** The ideal treatment for patients who survive from acute vertebrobasilar artery occlusion but develop aggressive ischemic events despite maximal medical therapy in the early non-acute stage is unknown. This paper reports the technical feasibility and outcome of staged endovascular treatment in a series of such patients with symptomatic intracranial vertebral artery occlusion.

**Methods:** Ten consecutive patients who presented with aggressive ischemic events in the early non-acute stage of intracranial vertebral artery occlusion from Jan 2015 to Nov 2020 were retrospectively reviewed. Among them, eight male and two female patients with a mean age of 66.7 years developed aggressive ischemic events, and the NIHSS score was elevated by a median of 7 points despite medical therapy. All patients received staged endovascular treatment 4–21 days from onset, at an average of 11 days. The strategy of staged treatment was as follows: first, a microwire was passed through the portion of the occlusion, which was then dilated with balloon inflation to maintain the perfusion above TICI grade 2b. Then, with the use of antiplatelet drugs, the residual intravascular thrombus was gradually eliminated by the continuous perfusion and an activated fibrinolytic system, leaving the residual stenosis. A second stage of angioplasty with stent implantation was subsequently performed if residual stenosis was ≥50%. The NIHSS scores and mRS scores were compared between pre- and post-endovascular treatment groups and in the follow-up period.

**Results:** Technical success was achieved in 9 patients who received staged endovascular treatment (perforation occurred in one patient during the first stage). The NIHSS scores were significantly improved, with a median score 7 points lower on discharge compared with the scores for the most severe status. Favorable outcomes with mRS score ≤ 2 were achieved in 7 and 9 patients at the 3-month follow-up and the latest follow-up, respectively, which was better than the preoperative status.

**Conclusion:** Staged endovascular treatment might be a safe, efficient, and viable option in carefully selected patients with symptomatic intracranial vertebral artery occlusion in the early non-acute stage. However, this needs to be confirmed by further investigation, preferably in a large, controlled setting.

## Introduction

Acute vertebrobasilar artery occlusion is a serious condition with high mortality rates that range from 80 to 95% (1). Thrombolysis and/or mechanical thrombectomy has historically achieved rates of recanalization of 45–78%, which provides the patients with favorable functional outcomes (2). However, effective therapies for acute treatment of ischemic stroke rely on timely restoration of the blood supply to salvageable brain tissue. Currently, only 10–20% of stroke patients in China reach the hospital within 3 h of the stroke onset (3). In addition, fewer than 3% of patients receive intravenous thrombolysis, a proportion much lower than that in high-income countries (4). A subset of patients with vertebrobasilar artery occlusion survive the acute events without endovascular recanalization due to the leptomeningeal collaterals; however, as the collaterals usually fail to provide sufficient perfusion during periods of increased oxygen demand, the patients may develop recurrent or aggressive ischemic events and progressive disability in the early non-acute stage (>24 h) despite intensive medical therapy. These patients miss the optimal period for endovascular recanalization, and extracranial-intracranial bypass treatment is not recommended in such cases because of technical challenges and a high risk of morbidity and mortality (5, 6). Herein, we report our experience with staged endovascular treatment in such patients.

## Methods

### Patient Enrollment

We retrospectively reviewed our maintained neurointerventional database for cases from January 2015 to November 2020 at Peking University First Hospital, Beijing. We identified consecutive cases of the intracranial vertebral artery occlusion in the early non-acute stage in our database. According to the results of the DAWN clinical trials, the time window of thrombectomy can be extended to 24 h (7), and the thrombosis is almost organized about 1 month after the occlusion (8), therefore, we defined the “early non-acute stage” as the time from 24 h to 1 month after the onset of ischemic stroke. Ten patients receiving endovascular treatment in this stage were enrolled in this study, including 8 males and 2 females with a mean age of 66.7 years (range: 52–78 years) (Table 1). All patients had high-risk factors for stroke (Table 1), and they all suffered typical ischemic symptoms of posterior circulation, such as vertigo, ataxia, dysarthria, blurred vision, facial paralysis, dysphagia and weakness. The National Institute of Health stroke scale (NIHSS) score at onset ranged from 3 to 11, with a median of 6. None of these patients had undergone intravenous thrombolysis or mechanical thrombectomy in the acute phase of infarction. Magnetic resonance imaging (MRI) or computed tomography (CT) examinations showed new or recent infarctions in the territory of the vertebrobasilar artery. All patients received CT angiography (CTA) or magnetic resonance angiography (MRA) examination, which confirmed that the dominant intracranial vertebral artery was occluded and that the contralateral vertebral artery was occluded, hypoplastic, or severely stenotic. Although intensive medical therapy and strict control of risk factors were used, these patients experienced elevated NIHSS scores and severe neurological manifestations such as disturbance of consciousness and hemiplegia. The NIHSS scores were 4–10 points higher in the most severe stage than at onset, with a median increase of 7 points. Because of the aggravation of symptoms despite extensive medical treatment, these patients ultimately received neuroendovascular treatment. The time between symptom onset and endovascular recanalization ranged from 4 to 21 days, with an average of 11 days (Table 1). Each patient himself or his or her family members were authorized to sign an informed consent form, and the protocol for this recanalization study was approved by our institutional ethics committee (No. G202037).

**Table 1:** Baseline characteristics of the enrolled patients.

**Case no**.	**Age(yrs)/Sex**	**Clinical presentation**	**Lesion location**	**NIHSS 1**	**Risk factors**	**Time 1, days**	**NIHSS 2**	**Findings of MRI after onset**
1	64/M	Vertigo, tinnitus, dysarthria, weakness	Lt VA	6	Hypertension, diabetes, myocardial infarction, hyperlipemia, smoking	21	10	Infarction in pons and bilateral cerebellum
2	72/M	Vertigo, dysphasia, facial paralysis	Rt VA	3	Diabetes	16	8	Fresh infarction in right cerebellum and medulla
3	52/F	Vertigo, ataxia, dysphasia, facial paralysis	Rt VA	4	Hypertension, diabetes	17	11	Infarction in right cerebellum
4	66/M	Vertigo, tinnitus, ataxia, weakness	Rt VA	8	Hypertension, hyperlipemia	9	18	Infarction in right cerebellum and pons
5	59/F	Vertigo, weakness, ataxia	Lt VA	10	Hypertension, diabetes, obesity	8	14	Infarction in bilateral cerebellum and pons
6	64/M	Vertigo, ataxia, facial paralysis	Rt VA	5	Hypertension, smoking	9	9	Fresh infarction in right cerebellum
7	70/M	Vertigo, ataxia, weakness, dysphasia	Rt VA	11	Hypertension, smoking, drinking, hyperhomocysteinemia	10	16	Fresh infarction in pons and bilateral cerebellum
8	73/M	Vertigo, ataxia, facial paralysis	Lt VA	4	Hypertension, diabetes	6	11	Fresh infarction in left cerebellum and pons
9	78/M	Dizziness, blurred vision, weakness	Rt VA	3	Smoking, drinking	10	13	Fresh infarction in right occipital lobe and cerebellum
10	69/M	Vertigo, weakness	Lt VA	7	Obesity, drinking, hyperlipemia	4	16	Fresh infarction in bilateral cerebellum and pons

*NIHSS 1, NIHSS score of onset; NIHSS 2, Worst NIHSS score in aggravation; Time 1, Time between symptom onset and recanalization; F, female; Lt, left; M, male; MRI, magnetic resonance imaging; Rt, right; VA, vertebral artery*.

### Strategy of Endovascular Treatment

#### Inclusion and Exclusion Criteria

The inclusion criteria for recanalization treatment were as follows: (1) patient age >18 years and <80 years; (2) the patient had evidence of infarctions related to intracranial vertebral artery distribution on MRI; (3) the patient was in an early non-acute stage of intracranial vertebral artery occlusion; (4) the unilateral or dominant intracranial vertebral artery was completely occluded together with total occlusion, hypoplasia, absence, or severe (≥70%) stenosis of the contralateral vertebral artery; (5) the patient had severe neurological manifestations refractory to maximal medical therapy, including double antiplatelet therapy plus a statin use and management of risk factors (e.g., smoking, drinking, diabetes, hyperlipidemia and hyperhomocysteinemia); (6) the patient had at least one atherosclerotic risk factor; and (7) the grade of collateral flow was ≤3 (according to the modified grading system of the American Society of Interventional and Therapeutic Neuroradiology) (9).

The exclusion criteria were as follows: (1) non-atherosclerotic occlusion, e.g., arterial dissection or vasculitis; (2) intracranial hemorrhage on CT or MRI; (3) known allergies or contraindication to aspirin, clopidogrel, heparin, tirofiban, contrast medium, metal, etc.; (4) life expectancy <1 year due to other diseases, such as malignant tumors; and (5) other conditions that were unconducive to general anesthesia or surgery.

#### Neurointerventional Procedure

All patients were scheduled to receive two stages of endovascular treatment. In the first stage, the procedure was performed under local anesthesia. Heparin was given as an anticoagulant throughout the procedure in all cases to maintain an activated clotting time between 250 and 300 s. Cerebral angiography was first performed to evaluate the condition of all cerebral arteries and collateral perfusion. After confirmation of the occlusive lesion, which usually included the V3 and V4 segments of the vertebral artery, a 6F guiding catheter (Envoy, Cordis, USA) was placed in the distal V2 segment. Then, an Echelon-10 microcatheter (Medtronic, USA) or an Excelsior SL-10 microcatheter (Stryker Neurovascular, USA) with the support of a Pilot-50 microwire (Abbott, USA) or a Synchro 14 microwire (Stryker Neurovascular, USA) was advanced through the occlusive lesion. Once injection confirmed that the microcatheter was in the true lumen of the distal part, a 300-cm-long Transend 300 Floppy microwire (Stryker Neurovascular, USA) was advanced to replace the microcatheter. Then, a Gateway angioplasty balloon (Stryker Neurovascular, USA) was placed at the site of the occlusion and inflated from the distal part to the proximal portions. Anterograde flow through the occluded portion was graded using the Thrombolysis in Cerebral Ischemia (TICI) grading system (10). Technical success in this stage was determined by recanalization with a TICI grade of ≥2b. If the TICI grade was <2b or the perfusion did not remain stable for over 30 min during the observation, one or more additional balloons could be used and inflated in the same way until the TICI grade was ≥2b; meanwhile, a bolus dose of tirofiban was injected through the guiding catheter, and then additional tirofiban was continuously infused intravenously for 48 h. Throughout the treatment process, we communicated with the patients to monitor their discomfort and conducted timely physical examinations if necessary.

After the first stage of endovascular treatment, a head CT scan was performed in the first 24 h after intervention. Intravenous injection of tirofiban was routinely used for 48 h, and preoperative drugs were maintained. Blood pressure was controlled below 140/90 mmHg. Follow-up CTA, MRA or transcranial Doppler (TCD) was performed once a week after the recanalization procedure. If the thrombus in the intracranial vertebral artery was eliminated and the residual stenosis was more than 50% or if there was a dissection in the portion with stenosis, a second stage of the neurointerventional procedure was performed. Nine patients with residual stenosis <50% received a second stage of endovascular treatment, and one patient had complication of dissection. The average interval between the two endovascular treatments was 15.6 d (11–25 d) (Table 2).

**Table 2:** Clinical summary of 9 patients undergoing staged neuro-endovascular recanalization.

**Case No**.	**mRS 1**	**First stage of neuro-endovascular recanalization**					**Time3, days**	**Second stage of treatment**				**mRS 2**	**NIHSS 3**	**mRS 3**	**ISR**	**mRS 4**
		**Angiographic findings**	**Collateral blood flow**	**Technical success**	**TICI grade**	**Intraoperative complication**		**Preoperative residual stenosis**	**Stent used (mm)**	**Technical success**	**Complications**					
1	4	V3 and V4 of Lt VA occlusion	Rt PCOM supplied PCA with retrograde flow to the top of the BA	Yes	2b	NA	15	80%	Apollo 3.5*18	Yes	NA	3	4	1	0	0
2	4	V4 of Rt VA occlusion	Lt PCOM supplied PCA with retrograde flow to the top of the BA	Yes	2b	NA	14	60%	Apollo 3.5*18	Yes	NA	3	3	1	-	1
3	4	V4 of Rt VA occlusion	Occluded Lt VA with leptomeningeal collaterals to BA	Yes	2b	VA dissection	25	75% (dissection)	Winspan 3.0*20	Yes	NA	2	2	0	50%	0
4	5	V3 and V4 of Rt VA occlusion	Lt PCOM supplied PCA with retrograde flow to the top of the BA	No	0	Microguidewire- perforated out of the VA						5	14	6		
5	4	V3 and V4 of Lt VA occlusion	Rt thin VA with severe stenosis in V4 segment	Yes	2b	NA	19	70%	Winspan 3.0*20	Yes	NA	3	4	1	-	1
6	4	V4 of Rt VA occlusion	Anastomosis between left ACA and distal VA	Yes	2b	NA	16	80%	Apollo 2.5*13	Yes	SAH	4	8	3	20%	2
7	5	Distal V2 to V4 segment of Rt VA occlusion	PCOMs supplied PCAs with retrograde flow to the top of the BA	Yes	2b	NA	15	60%	Apollo 2.5*13	Yes	NA	4	7	2	0	1
8	4	V4 of Lt VA occlusion	Rt hypoplasia VA with severe stenosis in V4 segment	Yes	2b	NA	11	70%	Winspan 3.5*20	Yes	NA	3	4	2	20%	1
9	4	V3 and V4 of Rt VA occlusion	Lt PCOM supplied PCA with retrograde flow to the top of the BA	Yes	2b	NA	13	60%	Apollo 3.5*13	Yes	NA	3	5	2	0	1
10	5	V3 and V4 of Lt VA occlusion	Rt hypoplasia VA with severe stenosis in V4 segment	Yes	2b	NA	12	70%	Appollo 3.5*18	Yes	NA	4	5	3	10%	2

*mRS 1, mRS score before the first stage of neuro-endovascular treatment; mRS 2, mRS score at discharge; mRS 3, mRS score at 3 months follow-up; mRS 4, mRS score at the latest follow-up; NIHSS 3, NIHSS score at discharge; Time3, The time between the first and the second stage of neuro-endovascular treatment; ACA, ascending cervical artery; BA, basilar artery; FU, follow-up; ISR, in-stent restenosis; Lt, left; mRS, modified Rankin Scale, NA, No complication; PCOM, posterior communication artery; PCA, posterior cerebral artery; Rt, right; SAH, subarachnoid hemorrhage; VA, vertebral artery*.

**Figure 1:**
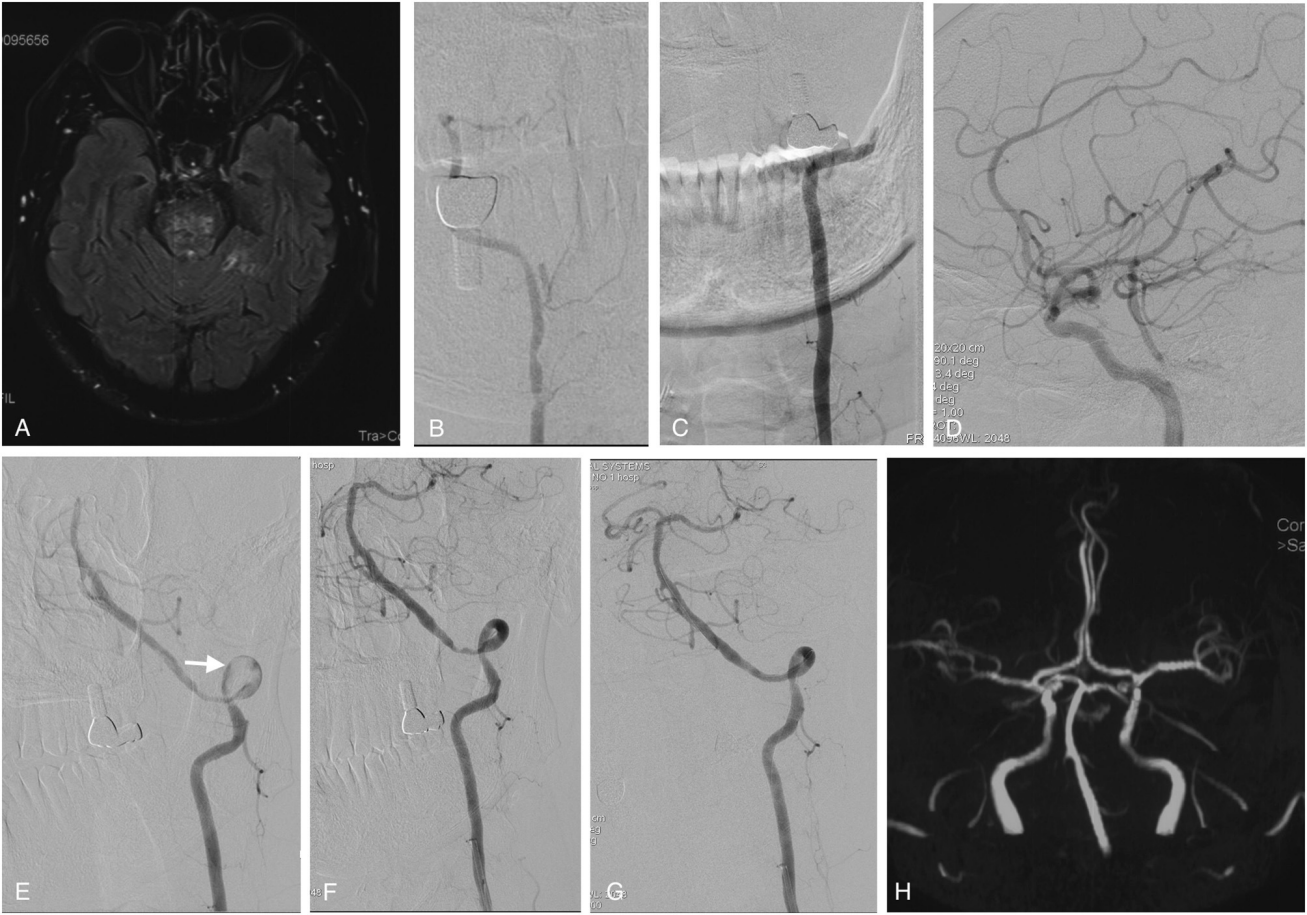
Imaging studies of case 1. Axial FLAIR MRI sequences showed ischemic infarctions in the pons **(A)** and bilateral cerebellum (not shown here). The right vertebral artery was hypoplastic **(B)**, and the left vertebral artery was occluded from the V3 segment onward **(C)**. The right PCOM supplied the PCA with retrograde flow to the top of the BA **(D)**. In the first stage of endovascular treatment, the left intracranial vertebral artery was recanalized by passing a microwire through and dilating a balloon in the stenotic region; some thrombus material remained on the vessel wall (white arrow) **(E)**. In the second stage of endovascular treatment, vertebral angiography showed that the thrombus had disappeared, leaving only the primary stenosis **(F)**, which was fully resolved by angioplasty with stenting **(G)**. Follow-up MRA performed 6 months later showed that the perfusion of the left intracranial vertebral artery and BA was unobstructed **(H)**. MRI, magnetic resonance imaging; FLAIR, fluid-attenuated inversion recovery; PCOM, posterior communicating artery; PCA, posterior cerebral artery; BA, basilar artery; MRA, magnetic resonance angiography.

In the second stage of endovascular treatment, the procedure was performed under general anesthesia. A 6F guiding catheter was placed in the V2 segment of the vertebral artery, and then a microwire was advanced through the stenotic lesion. A Gateway balloon was placed in the lesion and inflated, and then a balloon expanding Apollo stent (MicroPort Medical, Shanghai, China) or a self expanding nitinol Wingspan stent (Stryker Neurovascular, USA) was released across the stenotic lesion. Post-balloon inflation was performed if there was more than 40% residual stenosis. After the second stage of neurointerventional therapy, dual antiplatelet drugs were maintained for at least 6 months, and aspirin alone was given every day thereafter. Rehabilitation treatment was recommended for patients with functional disability (Illustrative case in Figure 1).

### Follow-Up Study and Statistical Analysis

All patients were followed up regularly. The modified Rankin Scale (mRS) and NIHSS scores were applied before endovascular treatment, at discharge and at follow-up. CTA, MRA or cerebral catheter angiography was performed at the 6-month or 1-year follow-up.

SPSS 16.0 statistical software was used for analysis. The Wilcoxon signed rank test was used to compare the NIHSS and mRS scores before the operation, at discharge and during the follow-up. The difference was statistically significant if *P* < 0.05.

## Results

**Figure 2:**
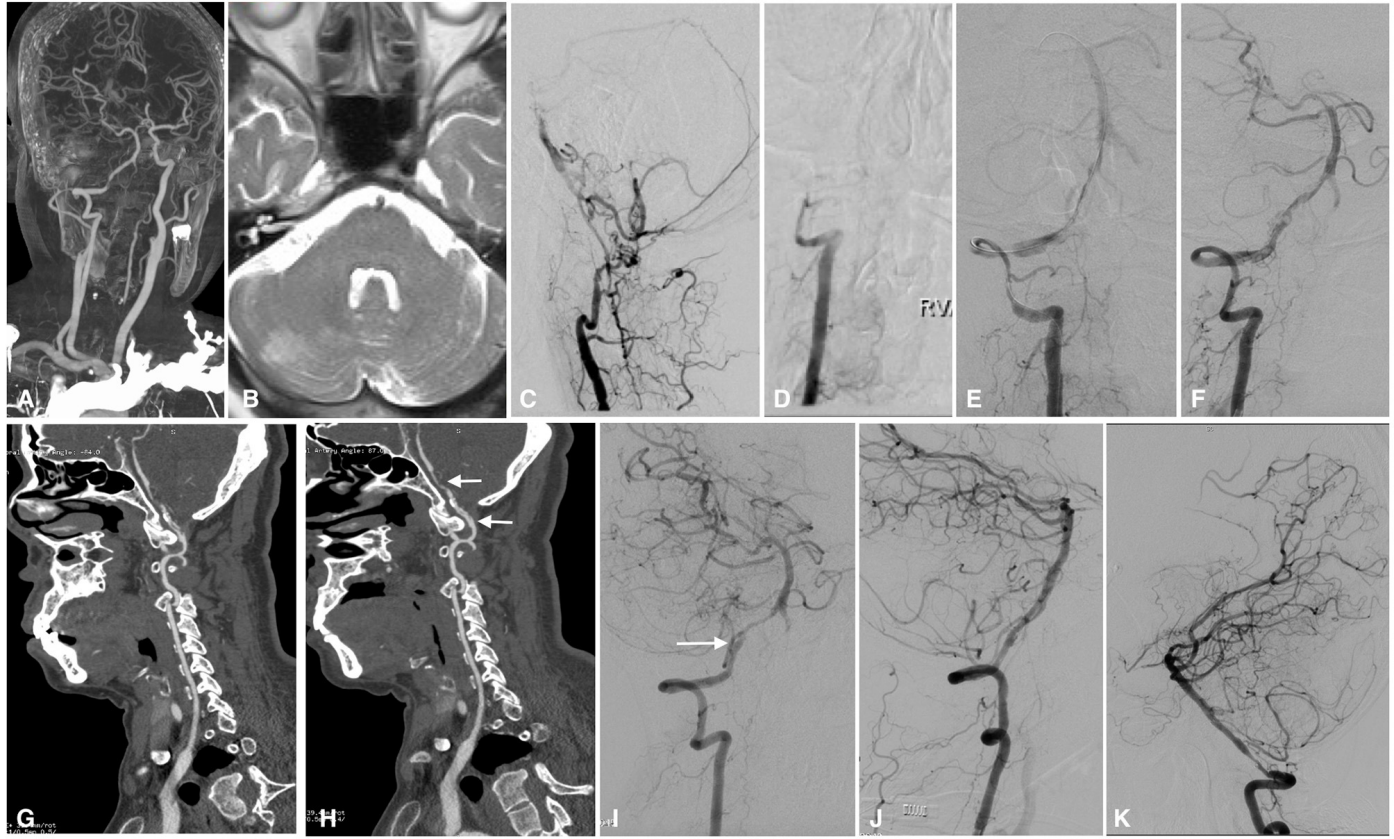
Imaging studies of case 3. CTA in a local hospital showed severe stenosis of the right intracranial vertebral artery 6 months before admission **(A)**. Axial T2-weighted MRI showed ischemic infarction in the right cerebellum **(B)**. The left intracranial vertebral artery was occluded, and the BA was supplied by the anastomotic branches and leptomeningeal collaterals **(C)**. The right intracranial vertebral artery was occluded from the V3 segment onward **(D)**. In the first stage of endovascular treatment, the right intracranial vertebral artery was recanalized by balloon inflation **(E)**, and stable perfusion was achieved after sufficient dilation with 3 balloons; however, some thrombus remained on the vessel wall **(F)**. CTA performed 1 week later showed intraluminal thrombus and a dissection in the right intracranial vertebral artery **(G)**. Two weeks later, CTA showed that the thrombus had decreased significantly, leaving a dissection and residual stenosis **(H)**. Angiography performed in the second stage confirmed that the thrombus had disappeared and that there was a dissection (white arrow) and residual stenosis in the V4 segment of the intracranial vertebral artery **(I)**; the dissection and stenosis were resolved by a Winspan stent **(J)**. Follow-up angiography performed 12 months later showed moderate stenosis in the proximal part of the stent **(K)**. CTA, computed tomography angiography; MRI, magnetic resonance imaging; BA, basilar artery.

Cerebral catheter angiography showed that all the 10 patients suffered occlusion of the intracranial vertebral artery on the dominant side. The occlusion sites included the V4 segment of the vertebral artery in 4 patients, originating at the V4 segment and extending to the V3 segment in 5 patients, and originating at the V4 segment and extending to the V2 segment in one patient. Collateral perfusion of the posterior circulation commonly came from the posterior communicating arteries (PCOMs) (5/10) and hypoplastic or stenotic contralateral vertebral arteries (3/10). Technical success of recanalization during the first stage was achieved in 9 cases, in whom the final perfusion was achieved at least on TICI 2b. The procedure failed in 1 patient (case 4) due to microwire perforation of the vertebral artery. Although the patient didn't suffer a symptomatic deterioration after the operation, he died of pneumonia and respiratory failure 3 months after discharge. Another patient (case 3) experienced asymptomatic dissection of the intracranial vertebral artery after the first stage of endovascular treatment (Illustrative case in Figure 2). The mean interval time between the two stages of neuroendovascular treatment was 15.6 d (11–25 d), and during which there were no hemorrhagic complications. Angiographic examination revealed that the residual thrombi in the vertebral arteries were all eliminated, leaving severe stenosis (>50%) in all patients. Then, the patients underwent angioplasty with stent implantation, during which nine stents were implanted, including 6 balloon-expanded Apollo stents and 3 self-expanding Wingspan stents. One patient (case 6) experienced aggressive headaches and disturbed consciousness after stent implantation, and a CT scan showed subarachnoid hemorrhage (SAH) around the pons and medulla associated with moderate hydrocephalus. The patient recovered well with ventricular drainage and rehabilitation. Among the 10 patients, symptoms and NIHSS and mRS scores had improved in 9 at the time of discharge. The NIHSS scores were significantly improved by a median of 7 points on discharge compared with the scores for the most severe status (*P* < 0.05). The patients were followed up for a mean of 12.8 months (range: 5–36). Excluding the patient who died (case 4), the median mRS scores were 2 (range: 0–3) at the 90-day follow-up and 1 (range: 0–2) at the latest follow-up, both of which were much better than the median score before the first stage of endovascular treatment (range: 4–5; median: 4) (*P* < 0.05). Angiographic follow-up was available for 7 patients, most of whom had no or mild in-stent stenosis (Table 2).

## Discussion

Acute vertebrobasilar artery occlusion is a life-threatening condition and requires immediate treatment. Despite recent advances in stroke care, the rate of poor outcomes for those with vertebrobasilar artery occlusion remains high (11). In 2016, a meta-analysis of 5 recent landmark endovascular therapy trials confirmed the superiority and better outcomes of mechanical thrombectomy in large-vessel occlusion for acute anterior circulation strokes (12). Several subsequent trials showed that high rates (ranging from 79 to 96.86%) of successful reperfusion and favorable outcomes could also be achieved in acute vertebrobasilar artery occlusion patients (13–15). Subsequent studies also showed that the window for thrombectomy in cases of acute ischemic stroke could be carefully extended to 24 or 16 h (7, 16, 17). However, there are still limited studies reporting on the ideal treatment strategy in patients presenting more than 24 h from stroke symptom onset. Some patients survived the acute stage of vertebrobasilar artery occlusion because of the collateral flow but developed aggressive ischemic events and progressive disability in the following stage despite receiving maximal medical therapy. A prior study by our group has reported carotid-vertebral artery bypass with saphenous vein grafts for symptomatic V1 segment occlusion (18). However, for patients with intracranial vertebral artery occlusion, bypass surgery is risky and technically challenging (5). Although it has been reported that multiple stenoses or occlusions in the posterior circulation might hide an underlying inflammatory vascular disease, which might increase the interventional risk (19), some researchers have attempted to recanalize the occluded intracranial vertebral artery with endovascular therapy in the non-acute stage.

There is limited literature reporting on interventional therapy for large occluded intracranial vessels beyond the acute phase (1, 2, 20, 21). In a recent study, Gao et al. summarized six previous studies on endovascular recanalization for non-acute occlusions after 2009 (21). In the seven reported studies, including the cases reported by Gao et al. there were 72 patients with early non-acute (6) or chronic (66) occlusion of the intracranial vertebral artery or basilar artery who received endovascular treatment, and the success rate of the technique was 93% (67 in 72 cases), with a mortality rate of 1.39% (1 in 72 cases). In our case series, the technical success rate was 90%, and the mortality was 0% at discharge. Complications occurred in 2 patients: asymptomatic intracranial vertebral artery dissection in one patient and subarachnoid hemorrhage in the other patient (case 6), which might be associated with heavy calcification in the intracranial vertebral artery because a recent study showed that larger vertebrobasilar artery calcification volumes were a significant predictor of reduced technical success and functional independence and increased mortality (22). Although our results seemed encouraging, it should be emphasized that revascularization of intracranial vertebral artery occlusion in the early non-acute stage is a high-risk procedure.

The major technical challenge during the first stage of endovascular recanalization is traversing the occlusion site with a microwire. Gao et al. indicated that preoperative high-resolution MRI (HRMRI) and simultaneous two-vessel injection during the recanalization procedure might be helpful in guiding the wire to the distal cavity (21). HRMRI is helpful in the diagnosis of intracranial vertebral artery occlusion and luminal thrombosis and can help to identify the subset of patients with high embolism risk before the procedure (23). Simultaneous two-vessel injection is a good technique for helping the surgeon advance the microwire to the true lumen of the distal vessel. In our case series, this technique was not conducted because the procedure of recanalization procedure was performed under local anesthesia, as slight movements make the road map images unclear. It is critical to communicate with the patient frequently during the procedure. If the patient complains of pain, sweating, nausea, or vomiting, it usually indicates that the microwire has penetrated the blood vessel wall. In our case series, there were two patients (case 3 with dissection and case 4 with perforation) with intraoperative complications. Both of these patients complained of pain as the microwire was being advanced through the occlusion, and sweating and vomiting occurred in case 4. We suspect that patients' self-reports might be more accurate and occur earlier than indications from angiography in reflecting whether the microwire is in the true lumen.

Treatment of early non-acute thrombi is the key to recanalization. In the acute stage, it is advisable to use recombinant tissue plasminogen activator for thrombolysis or mechanical thrombectomy because the thrombus is not firm. In the chronic stage, as the thrombus is well-organized and strongly adhered to the vessel wall, the key point of recanalization is to pass the microwire through the occluded lesion, and there is no need to deal with the organized thrombus except for performing balloon inflation. There are no reports in the literature regarding how to deal with an intravascular thrombus in the early non-acute stage of occlusion, as recanalization treatment is a relatively contraindicated in this stage, and there is a considerable risk of hemorrhagic conversion with early revascularization. In our previous cases involving anterior circulation, we attempted thrombectomy with aspiration and a stent retriever, which failed because the thrombus had a gum-like consistency. New-generation aspiration devices with larger bores and stronger suction force might be useful in clearing the thrombus from the proximal part of the original stenosis (24); however, we speculate that there could be many thrombi in the distal part of the stenoses due to the absence of continuous perfusion, and the aspiration catheter should not be placed over the original stenotic part to aspirate the distal thrombus as this would be dangerous and might result in dissection or thrombus translocation. In our research, we found that the thrombus in the early non-acute stage could be inflated by a balloon without translocation, and the anterograde flow could be achieved stably. Furthermore, these thrombi could be slowly eliminated through sustained blood flow, an activated human fibrinolytic system, and antiplatelet drugs. Among the 9 patients undergoing staged treatment, thromboembolisms did not occur during our observation, suggesting that thrombus elimination with drugs and the patient's own fibrinolytic system is safe. CTA or MRA can clearly show the intraluminal structure, including the dissection and thrombolysis (25–27). Through weekly CTA or MRA examination, we observed that the time to complete thrombus elimination was ~2 weeks, depending on the length of the thrombus, the time from occlusion, the degree of perfusion recovery and the response of patients to antiplatelet drugs. After thrombus elimination, angioplasty with stenting in the severe residual stenotic area becomes straightforward.

Although staged treatment increases the length of hospitalization, our strategy still has some advantages. First, after the initial intervention treatment, there is usually moderate to severe residual stenosis due to the residual thrombus, which limits the perfusion to the basilar artery and reduces the risk of hemorrhagic conversion. Second, in the later stage, the stent does not need to be implanted if the residual stenosis is not severe or only one stent needs to be deployed in the residual severely stenotic portion; thus, it is not necessary to use multiple stents to cover the thrombus, as it would be if only one stage of treatment were performed. Third, our strategy avoids the deployment of stents in the atlantoaxial segments of the vertebral artery, where stents may be develop fractures or in-stent occlusion in the long term as a result of cervical movement. Finally, our study showed good outcomes with an acceptable rate of technical success and a low rate of severe complications.

There are also many limitations in our study. First, the number of subjects was small (*n* = 10). Future studies with a greater number of included subjects could provide more persuasive evidence. Second, this is a retrospective study and lacks a control group, and therefore, it does not definitively show that staged intervention is more beneficial than other treatment strategies. Third, after revascularization in the first stage, we injected a loading dose of tirofiban into the blood vessel and maintained it for 48 h. Although recent research conducted by Quan et al. showed that low-dose tirofiban did not increase the risk of symptomatic intracranial hemorrhage or 90-day mortality in endovascular treatment of acute intracranial vertebrobasilar artery occlusion (15), its safety, effectiveness and necessity in the early non-acute stage of occlusion recanalization need to be further confirmed. Forth, some recent studies have shown that simultaneous endovascular recanalization and stent implantation may be safe and effective for such patients (23). However, how to select these patients and avoid the risks of hemorrhagic transformation needs further research. Fifth, in this study, we used TICI grading as a measure of technical success for posterior circulation, which is mostly commonly used for anterior circulation. Although some other authors have also used this grading system, AOL might be better in evaluating the posterior circulation (23, 28). Sixth, NIHSS is not ideal to assess stroke in the posterior circulation, because several manifestations of stroke in the posterior circulation, such as vertigo, vomiting, gait instability, and truncal ataxia, are not represented on the NIHSS scale (29). Furthermore, the several angiographic examinations of CTA or catheter angiography increase the risk of renal injury in patients. Finally, as there was variability in the types of stents used (balloon-mounted and self-expanding stents), the durability of stents in this context is also unclear and needs to be further studied. However, our results are encouraging, with a high recanalization rate and a low rate of significant intracranial hemorrhage. Our strategy may be considered an option in a highly select group of patients who have aggressive ischemic events or symptoms despite maximal medical therapy. The effectiveness of this approach in the prevention of progressive strokes and in the improvement of long-term outcomes should be evaluated prospectively in the future.

## Conclusions

Our report based on a small case series suggests that staged neuroendovascular treatment of intracranial vertebral artery occlusion in the early non-acute stage is feasible, with an acceptable rate of technical success and a low rate of complications. However, its efficacy and safety need further investigation, preferably in a randomized controlled setting.

## Data Availability Statement

The raw data supporting the conclusions of this article will be made available by the authors, without undue reservation.

## Ethics Statement

The studies involving human participants were reviewed and approved by Peking University First Hospital ethics committee. The patients/participants provided their written informed consent to participate in this study.

## Author Contributions

HD: conception and design. CL, ZY, and YZ: acquisition of data. HD and LC: drafting the article. JZ: critically revising the article. SS, YW, and LL: technical support. All authors contributed to the article and approved the submitted version.

## Conflict of Interest

The authors declare that the research was conducted in the absence of any commercial or financial relationships that could be construed as a potential conflict of interest. The Handling Editor declared a shared affiliation, though no other collaboration, with one of the authors LC.
